# INVESTIGATING THE BUILT ENVIRONMENT SURROUNDING NATURALLY OCCURRING RETIREMENT COMMUNITIES (NORCS) IN TORONTO

**DOI:** 10.1093/geroni/igae098.3862

**Published:** 2024-12-31

**Authors:** Laura Fusca, Paula Rochon, Tai Huynh, Shoshana Hahn-Goldberg, Lavina Matai, Longdi Fu, Rachel Savage

**Affiliations:** University of Toronto, Mississauga, Ontario, Canada; Women’s College Hospital, Toronto, Ontario, Canada; University Health Network, Toronto, Ontario, Canada; University Health Network, Toronto, Ontario, Canada; ICES, Toronto, Ontario, Canada; ICES, Toronto, Ontario, Canada; Women’s College Hospital, Toronto, Ontario, Canada

## Abstract

A positive built environment can improve the health of older adults and support aging in place by increasing access to amenities, physical activity, and social interaction. We investigated the walkability and amenity density surrounding Naturally Occurring Retirement Communities (NORCs) in Toronto, areas where many older adults live. This population-based descriptive study linked two built environment datasets with a provincial registry of high-rise NORC buildings in Ontario, Canada by postal code (PC). Mean (SD) walkability index scores and quartiles, as well as amenity density categories were compared for NORC and non-NORC PCs. Building and resident characteristics were also compared for NORC PCs in the least and most favourable categories for each outcome. Our analysis of walkability and amenity density included 49,295 (488 [9.9%] NORC) and 49,945 (489 [9.8%] NORC) Toronto PCs respectively. Walkability was significantly higher for NORC (mean 7.1 (SD 9.1)) versus non-NORC (4.9 (8.3)) PCs; although 57 (11.7%) NORC PCs were in low walkability neighbourhoods. NORC PCs were also more amenity dense, with 63.0% being classified as medium/high density compared to 50.5% of non-NORC PCs. Low walkability and amenity-poor NORCs tended to house older, immigrant, ethnically diverse and lower-income populations. Low walkability NORCs were also more likely to be apartments with supports and have residents without access to basic needs. Overall, our findings suggest that many NORCs are well-positioned to support aging in place given their walkability and amenity access, however action should be taken to support NORCs with suboptimal environment conditions.

